# Treatise on Diphtheria

**Published:** 1881-08

**Authors:** 


A TREATISE ON DIPHTHERIA; ITS HISTORY, ETIOLOGY, VARIETIES, PATHOLOGY, SEQUELÆ, DIAGNOSIS AND HOMŒOPATHIC THERAPEUTICS. By *A. McNeil, M. D.* pp. 145, price $1.00. Chicago: Duncan Bros. 1881.

This book received the prize of one hundred dollars offered by the publishers for the best essay on diphtheria. In his preface, the author says of the germ theory: "The careful experiments I have quoted, show conclusively that the theory is an unreliable hypothesis." Again: "There is but one way for us to travel, viz.: that surveyed and built by Hahnemann, to give *the*

remedy in the minimal dose that corresponds most nearly to the totality of the symptoms; no gargles, no swabs, no caustics, no specifics." Turning now to p. 36, we find that the author asks the question: "Are the bacteria, which exist in the diseased mucous membrane, and which penetrate even the entire system, the producers or the product of the disease?" In answering this question he has made a very intelligent collection of evidence tending to overthrow the germ theory. The distrust of this theory among the old school is becoming more general every year, as a careful perusal of the medical journals will show. There are not wanting surgeons of large experience who entirely repudiate "Listerism." The natural history of germ life is established beyond dispute; yet the bounds of ascertained fact are overstepped, and by theories built of analogy, the principles governing the existence and multiplication of germs in organic fluids *outside* of the organisms that formed those fluids are made to account for phenomena occurring in the organisms themselves.

Pasteur * teaches that the bacteria *feed* upon the organic fluids, extracting therefrom those elements that contribute to their own sustenance, whilst the remainder of the constituents of the fluid falls into a sort of ruin like an arch from which the keystone has been removed. This *débris* then re-arranges itself into new forms, such as water, carbonic acid, the various gases of putrefaction, various new forms of poisonous character; or, in certain cases, alcohol. If then such results follow the introduction of bacteria and other algæ into the above mentioned fluids, do the same results occur in those fluids which are still contained within the living organism (the blood, for example)? Assuredly not *if the organism be in a sufficiently healthful condition to resist them.* This view, once peculiar to Hahnemannians, has lately been adopted by a few leading men in the old school.

"It is still a question, however," says the learned Dr. Carpenter, in his celebrated book on the microscope (p. 10), "which has to be decided upon other than microscopic evidence, how far the attacks of these fungi are to be considered as the *causes* of the diseases to which they stand related, or whether their development (as is the case in many parallel instances) is the *consequence* of the previously unhealthy condition of the plants which they infest; the general evidence appears to the author to incline to the latter view, which does not exclude their injurious action." Again, on page 393 of the same work, the distinguished author gives evidence that *every* grain of wheat has adhering to it the germs of blight, which do not develop if the grain be perfectly healthy. If this view be correct, homœopathy is sustained. If it be wrong, then homœopathic treatment must fall with it; or, as expressed by Dr. McNeil (p. 44), "we would be compelled to resort to gargling, cauterization, and large doses of drugs, capable of destroying the fungi."

With regard to the department of therapeutics, it is to be regretted that there is no repertory to the remedies. Nothing adds so to the value of these little monographs as a good repertory. It is the secret of the success of Dr. J. B. Bell's now celebrated book on diarrhœa. Dr. McNeil should prepare such a repertory for his next edition. In the pathogenesis of *Lac. Can.* the

*See Pasteur's "Studies in Fermentation," translated by F. Faulkner & D. C. Robb, B. A MacMillan & Co.: London and New York, 1879.

exudation is represented as going from left to right, like *Lachesis*. This, we are inclined to think, is an error. The symptoms of *Lac. Can.* commence on the left side, then leave that side altogether and reappear on the right side. They soon get better on the right side, and return to the left again, and so on.* In the pathogenesis of *Lachesis*, third line from the bottom, should read, "fluids are swallowed with *more* pain than solids." The statement in the text reads *less*. This error originally appeared as a typographical blunder in Lippe's Materia Medica, from which it was probably inadvertently taken. There are a few typographical errors, as on page 37, line thirteen, the decimal point is placed to the right of the fraction. It should be at the *left*, thus: .001. On page 39, *Algæ* is misspelled. On the whole, this is an excellent little book, well worthy of the prize. We congratulate the committee who awarded it upon their excellent judgment.

W. M. J.

* See Dr. Swan's proving of *Lac. Can.* in *The Organon*, Vol. iii, p. 405, paragraph 3d.

